# The Effect of Antiresorptive Drug Holidays on Medication-Related Osteonecrosis of the Jaw: A Systematic Review and Meta-Analysis

**DOI:** 10.7759/cureus.30485

**Published:** 2022-10-19

**Authors:** Ali A Aboalela, Fathima Fazrina Farook, Amerah S Alqahtani, Mandlin A Almousa, Rehab T Alanazi, Duaa S Almohammadi

**Affiliations:** 1 Maxillofacial Surgery and Diagnostic Sciences Department, College of Dentistry, King Saud Bin Abdulaziz University for Health Sciences, Riyadh, SAU; 2 King Abdulaziz Medical City, Ministry of National Guard for Health Affairs, Riyadh, SAU; 3 King Abdullah International Medical Research Center, Ministry of National Guard for Health Affairs, Riyadh, SAU; 4 Preventive Dental Science Department, College of Dentistry, King Saud Bin Abdulaziz University for Health Sciences, Riyadh, SAU; 5 College of Dentistry, King Saud Bin Abdulaziz University for Health Sciences, Riyadh, SAU

**Keywords:** antiresorptive drug, tooth extraction, medication-related osteonecrosis of the jaw, osteonecrosis, mronj, drug holiday

## Abstract

The objective of this study is to evaluate the effectiveness of discontinuing high-dose antiresorptive (AR) therapy in reducing the risk of medication-related osteonecrosis of the jaw (MRONJ) in patients treated with AR medications and undergoing dentoalveolar surgery or tooth extractions.

The review was carried out in accordance with the Preferred Reporting Items for Systematic Reviews and Meta-Analyses (PRISMA) recommendations. A literature search was conducted using the databases MEDLINE, Embase, Web of Science, Scopus, and Cochrane Central Register of Controlled Trials (CENTRAL) from inception till the 1st of April, 2022. Both observational and interventional studies that evaluated the effect of AR drug holiday in the development of MRONJ in patients receiving AR medications and who require dentoalveolar surgical procedures were included. Trials published as abstracts, case reports, case series, non-systematic reviews, and others were excluded. All findings were reported as odds ratios (ORs) and corresponding 95% confidence intervals (CIs). The Newcastle-Ottawa Quality Assessment Scale was used to evaluate the methodological quality assessment, and the Grading of Recommendations, Assessment, Development, and Evaluations (GRADE) approach was used to evaluate the quality of the evidence.

Eight articles (6808 subjects) were included for analysis. Of the participants, 4847 cases (drug holiday group) were compared to 1961 controls (non-drug holiday group). Based on the random effects model, the pooled summary OR was 0.73 (95% CI: 0.51-1.06) for the drug holiday group compared to the non-drug holiday group. In other words, the drug holiday group was not significantly different from the non-drug holiday group in the development of MRONJ following a tooth extraction procedure (p = 0.10). The statistical heterogeneity was low across all studies (I^2^ = 13%, p = 0.33).

Within the limits of the available evidence, our findings revealed that drug holidays with AR will not minimize the risk of MRONJ and thus cannot be advised. It may be possible to arrive at more definitive conclusions from large prospective studies and randomized trials of good quality.

## Introduction and background

The medication-related osteonecrosis of the jaw (MRONJ) generally occurs when certain antiresorptive (AR) medicines commonly used to treat osteoporosis and cancer, such as bisphosphonates and denosumab, are used alone or in combination with antiangiogenics or immune modulators [1]. The American Association of Oral and Maxillofacial Surgeons (AAOMS) defines MRONJ as an exposed area of bone, or bone that can be probed through an intra- or extra-oral fistula that has persisted for more than eight weeks, in a non-irradiated jaw free of metastatic disease of a patient treated with AR alone or in combination with antiangiogenic or immune modulator agents [2]. MRONJ occurs rarely in people receiving low cumulative doses of these medicines. However, in people receiving these drugs at higher doses, specifically for cancer-related conditions, the risk of MRONJ may be higher and has been reported to occur in up to five in 100 individuals [3]. Based on the severity level, AAOMS has classified it into four stages.

The risk of MRONJ varies from low to high cumulative dosage, short to long duration of treatment, and frequency of administration of AR agents (osteoporosis versus cancer) [4]. Patients with cancer receiving high doses of AR drugs are therefore at greater risk [4]. Several risk factors have been identified for MRONJ, but the etiology and pathogenesis of the disease are still not fully understood and remain to be considered multifactorial. The most important independent risk factor for MRONJ is tooth extraction, according to numerous studies [5-7]. It is therefore recommended to avoid tooth extractions when patients are being treated with high doses of AR agents. An AR drug holiday may be recommended or considered if a tooth extraction is inevitable [7]. National guidelines or position papers in some countries recommend a drug holiday, but no international consensus has been reached regarding high-dose AR drug holidays [8-10].

A high-dose AR drug holiday remains uncertain in terms of its effectiveness in reducing the risk of MRONJ [11]. The absence of strong evidence regarding the efficacy of a high-dose AR drug holiday warrants this systematic review and meta-analysis evaluating the effectiveness of discontinuing high-dose AR therapy in reducing the risk of MRONJ in patients treated with AR medications and undergoing dentoalveolar surgery. Furthermore, trial sequential analysis (TSA) was performed to determine whether the currently available evidence is sufficient and conclusive.

## Review

This review paper was performed according to the Preferred Reporting Items for Systematic Reviews and Meta-Analyses (PRISMA) statement. The protocol was registered in the Open Science Framework (OSF) database (https://doi.org/10.17605/OSF.IO/GNY2W). The research question was formulated using the following Population, Intervention, Comparison, and Outcome (PICO) criteria: population: adults on AR (bisphosphonates or denosumab) drugs (oral or IV) and in need of dentoalveolar surgery or tooth extraction; intervention: drug discontinuation (i.e., drug holiday) of AR therapy (prior to and/or after) at the time of tooth extraction or dentoalveolar surgery; comparison: drug continuation (i.e., no drug holiday) of AR therapy (prior to and/or after) at the time of tooth extraction or dentoalveolar surgery; outcome: development of MRONJ (+/-).

The research question was whether an AR drug holiday will reduce the risk of MRONJ following a dentoalveolar surgical procedure/tooth extraction.

The databases MEDLINE, Embase, Web of Science, Scopus, and Cochrane Central Register of Controlled Trials (CENTRAL) were systematically searched from inception till the 1st of April, 2022 using the following search terms: (("antiresorptive*" or bisphosphonate* or alendronate or zoledronate or "zoledronic acid" or denosumab or risedronate or ibandronate or pamidronate or Zometa) AND (osteonecrosis or "avascular necrosis" or "jaw necrosis" or MRONJ or BRONJ or DRONJ or ONJ or "medication-related" or "bisphosphonate-related" or "denosumab-related")) AND ("Drug holiday" or cessation or discontinuation or interruption or stop or holiday or suspension or break).

We also searched two trial registers (ClinicalTrials.gov and World Health Organisation International Clinical Trials Registry Platform). We searched all studies that investigated the impact of AR drug holidays on the risk of MRONJ regardless of the measured outcomes. The search was restricted to English language articles and articles that are not written in English were only included if the journal provided an English-translated version. All the bibliographies of the chosen studies were hand-searched for additional studies. The authors were contacted at least twice if any data were missing. Case reports, case series, non-systematic reviews, in vitro and animal studies, and trials published as abstracts were excluded.

The inclusion criteria were any observational or interventional studies that evaluated the effect of AR drug holiday in the development of MRONJ in patients receiving AR medications and who require dentoalveolar surgical procedures.

The diagnosis of MRONJ was based on clear diagnostic criteria defined by AAOMS [2]. The primary outcome was the presence or absence of MRONJ. The following data items were collected from each literature source: citation, year of study, country of study, study design, population, primary disease, age groups, study intervention, sample size, type and duration of AR treatment, number of patients in drug holiday and comparison groups, duration of drug holiday, development of MRONJ, confounding factors, the authors’ suggested drug holiday recommendations, conclusions, and main results.

The titles and abstracts were independently reviewed for eligibility criteria by three authors (AA, MA, and RA). If a study was deemed unacceptable by all authors, the study was excluded. The fourth author was responsible for resolving any differences at this point (FF). The same three authors (AA, MA, and RA) independently screened the full texts of qualifying papers. For a final decision, any disagreements were discussed with the fourth author (FF).

All the studies were evaluated for risk of bias, using the Newcastle-Ottawa (NCO) Scale. Two authors independently did the Grading of Recommendations, Assessment, Development, and Evaluations (GRADE) evaluations of the quality of the evidence and the summary of findings (RA and DM). Any disagreements were resolved by the third author (FF). Five criteria were used to evaluate the quality of the evidence using the Cochrane Handbook (risk of bias, inconsistency, indirectness, imprecision, and publication bias).

Statistical analysis was performed using RevMan, version 5.3 (The Cochrane Collaboration, Copenhagen, Denmark). Funnel plot analysis was not performed, as fewer than 10 studies were included in the measured outcome. A test of heterogeneity was conducted using the I^2^ statistic. A value of 40%, 40-60%, or 60% was used to determine low, moderate, and substantial heterogeneity, respectively. A two-sided p-value of <0.05 was considered statistically significant for heterogeneity. Results were expressed as odds ratios with 95% confidence intervals (CIs). Using a random-effects model analysis (Mantel-Haenszel method), the estimates were pooled. We used sensitivity analysis to explain the diversity in the results from different studies using the following factors: primary disease, route of administration, and study effect as stratifying variables to identify any changes in the magnitude and direction of the statistical results. Further sensitivity analyses were carried out by sequentially removing individual studies (from the most recent trials) and re-analyzing the remaining dataset for outcomes with significant heterogeneity.

To assess the conclusiveness of the meta-analyses by providing more information on the precision and uncertainty of the results, the trial sequential analysis (TSA) was carried out using the Trial Sequential Analysis software version 0.9.5.5 beta (Copenhagen Trial Unit, Copenhagen, Denmark). Based on a 5% chance of type 1 error, 80% power, and a 20% relative risk reduction, the required information size (RIS) of the meta-analysis and adjusted significance levels were determined.

Results

**Figure 1:**
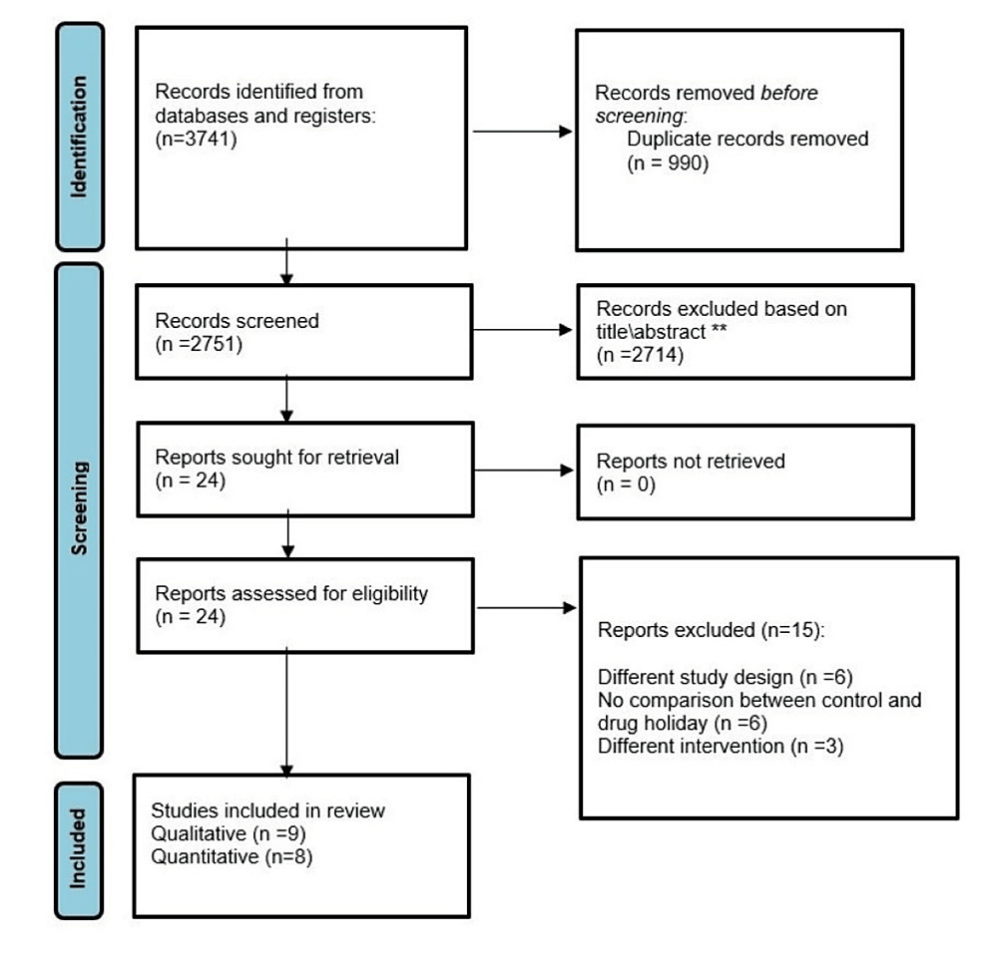
PRISMA flowchart demonstrating the reports identified, screened, and included in the review PRISMA: Preferred Reporting Items for Systematic Reviews and Meta-Analyses.

Our primary electronic search from PubMed, CENTRAL, Embase, Web of Science, and Scopus yielded 3741 citations. After the removal of the duplicates, 2751 citations were screened in terms of the title and abstract, and 25 articles were retrieved for full-text screening. After a critical review of the full texts, nine articles were identified as relevant for the qualitative synthesis and eight articles (6808 subjects) were included in the final review for the quantitative analysis (Figure 1). The PRISMA flowchart is presented in Figure 1.

We included nine studies in our review [12-20]. All studies were observational and one study was an interventional study [20]. Among the observational studies, two were prospective [12,16] and six were retrospective studies [13-15,17-19]. The characteristics of the included studies comprised descriptive data, such as study characteristics of the study population, type of ARs, duration of AR treatment, MRONJ characteristics, drug holiday characteristics, and the final outcome, and are summarized in Table 1. No potential conflict of interest was declared in the studies. One of the papers did not give the information directly and we contacted the authors to receive the information [13].

**Table 1:** Characteristics of included studies MRONJ: medication-related osteonecrosis of the jaw; BP: bisphosphonate; IV: intravenous; PO: oral administration; DH: drug holiday; AR: antiresorptive; BMA: bone modifying agents; ARONJ: antiresorptive agent-related osteonecrosis of the jaw; ECOG: Eastern Cooperative Oncology Group.

Author, year	Design	Population	Intervention	Primary disease	Types of antiresorptive (AR)	Duration of AR	Route of administration	Duration of drug holiday	# of control group	Outcomes of control group	# of intervention group	Outcomes of intervention group	Confounding factors assessed	Site	Conclusion
Hasegawa et al. (2013) [16]	Prospective, observational study	434 dental extraction, 201 patients (18 males and 183 females)	Tooth extraction	Osteoporosis	Alendronate, risedronate, alendronate/risedronate, minodronate	1-180 months	Oral	3 months	172 teeth	1 case confirmed stage 1 MRONJ (more than 120 weeks for primary healing	262 teeth	2 cases of delayed healing (3 months DH)	Duration of oral BP use + steroid therapy + anti-cancer therapy + immunosuppressive therapy + type of morbidity treated with BP	Not mentioned	The study supports the idea of a drug holiday
Bodem et al. (2015) [12]	Non-randomized, single-center, prospective study	102 extraction sites, 61 patients	Surgical tooth extraction\osteotomy	Caner\multiple myeloma	Zoledronic acid, ibandronate, pamidronate	40.25 months on average	IV	17.6 ± 15.9 months	17 Patients	1 case developed MRONJ	44 patients	7 cases developed MRONJ	Site of extraction, type of surgical intervention, anti-biotic prophylaxis, duration of AR drug discontinuation	47 sites mandibular, 55 maxillary sites	Patients currently undergoing intravenous BP therapy showed no higher risk for MRONJ compared with patients who have paused or completed their intravenous BP therapy, providing evidence that a drug holiday might not be considered in intravenous BP therapy
Hasegawa et al. (2017) [15]	Retrospective, multicenter, observational study	2458 dental extraction, 1175 patients (161 males and 1014 females)	Tooth extraction	Not mentioned	Alendronate, risedronate/minodronate, minodronate/others	1-246 months (mean ± SD: 38.5 ± 37.7)	Oral	3 months and 2 months	1200 site	MRONJ present in 30 sites and absent in 1170 sites	Drug holiday 3 months (1899 sites) 2 months (1818 site)	25 sites with MRONJ (3 months DH), 27 sites with MRONJ (2 months DH)	Smoking history + duration of oral BP + type of oral BP	27 mandibular (mand), 14 maxillary (max)	This study recommends a minimally traumatic extraction technique, removal of bone edges, and mucosal wound closure. The effectiveness of a short-term drug holiday was not confirmed as it has no significant impact on MRONJ incidence
Kawakita et al. (2017) [19]	Retrospective, multicenter, observational study	850 dental extractions, 402 jaws, 341 patients	Tooth extraction	Osteoporosis	2nd generation bisphosphonates or others	Discontinuing group mean (43.3 months), continuing group mean (31.3 months)	Oral	3 months before extraction and 2 weeks to 2 months after	118 jaws	None developed MRONJ	284 jaws	7 jaws with MRONJ	Diabetes + steroids therapy + malignancy + rheumatoid arthritis + renal failure + type of BP + duration of treatment + dental factors (No. of teeth extracted) + reason for extraction and site (upper or lower), wound status (open or complete closure)	4 mand, 3 max	Results suggest that discontinuing oral BPs does not prevent MRONJ after tooth extraction. We consider that oral BPs are not necessarily required to withdraw in patients with osteoporosis undergoing tooth extraction
Hasegawa et al. (2019) [14]	Non-randomized, multicenter, retrospective study	163 dental extraction, 85 patients	Tooth extraction	Cancer	Zoledronate, denosumab, zoledronate/denosumab, alendronate/denosumab, risedronate/denosumab	1-60 months	IV	Not mentioned	105 teeth	24 teeth (sites) developed MRONJ	58 teeth	17 sites with MRONJ	Smocking history + type of BMA + duration of BMA + pre-existing inflammation + surgical procedure-related factors (primary wound closure)	31 mand, 10 max	The effectiveness of a short-term drug holiday was not confirmed, as drug holidays had no significant impact on MRONJ incidence
Fujieda et al. (2020) [13]	Retrospective observational study	232 patients (125 autoimmune, 107 non-autoimmune)	Tooth extraction	Osteoporosis	Bisphosphonate (alendronate, risedronate, minodronate) or denosumab	Holiday group: 37 months (interquartile range: 14-69), continuation group: 41 months (interquartile range: 37-51)	Not mentioned	AR discontinued 3 months before the dental procedure	57 patients	3 patients develop osteonecrosis fracture	68 patient	7 patients with MRONJ	Age, sex, observation time, BMI, rheumatoid arthritis, glucocorticoid therapy, diabetes mellitus, malignant tumor, previous fractures, vitamin D, smoker, bisphosphonates (alendronate, risedronate, minodronate ibandronate, denosumab), administration period of AR	Not mentioned	Discontinuation of AR would not significantly contribute to reducing the incidence of ARONJ in those patients
Kang et al. (2020) [18]	Non-randomized, single-center, retrospective study	1323 dental extraction, 465 patients (45 males and 420 females)	Tooth extraction	Osteoporosis\cancer	Oral bisphosphonates (oral alendronate), ibandronate IV	Drug holiday: 53.3 ± 12.2 months, control: 65.2 ± 34.6 months	Oral and IV	39.0 ± 35.5 months	179 patients (537 teeth)	1 case of MRONJ	286 patients (786 teeth)	None	All patients, age, sex, duration of BP administration, duration of IV BP administration, duration of BP discontinuation, PO:PO + IV:IV, surgical extraction with osteotomy, osteoporosis:cancer, post-extraction MRONJ (patients who took BP for more than 3 years were analyzed separately using the same parameters)	Not mentioned	BP medication drug holiday is not recommended to reduce the risk of MRONJ
Hasegawa et al. (2021) [17]	Non-randomized, retrospective study	136 dental extraction, 72 patients (31 male and 41 females)	Tooth extraction	Cancer\multiple myeloma	Denosumab	Range: 1-85 months	Not mentioned	1 month	64 teeth	21 sites developed DRONJ	72 teeth	18 sites with MRONJ	Duration of denosumab drug holiday, additional surgical procedures, antibiotic administration, pre-existing inflammation	14 mand, 10 maxi, 1 both	Drug holidays for less than 9 months have no significant impact on the risk of MRONJ (in patients receiving high oncologic doses)
Ottesen et al. (2022) [20]	Parallel group, clinical, randomized, single-blind feasibility trial	31 dental extraction, 23 patients (11 males and 12 females)	Tooth extraction	Cancer	Denosumab, bisphosphonate	Denosumab = median: 9 months (range: 2-30 months)/ bisphosphonates = median: 17.5 months (range: 4-96 months)	IV or subcutaneous	1 month	10 patients	No MRONJ	13 patient	4 patients with MRONJ	Age, sex, type of cancer (breast, prostate, multiple myeloma), duration of AR medications, # on oral and IV chemotherapy, # on other medications, ECOG performance, previous or current tobacco user, development of MRONJ, patients’ self-reported health state measured with EuroQol (EQ)-5D-5L and the EQ-Visual Analog Scale (EQ VAS)	Not mentioned	All patients with cancer progression, as well as all 4 patients who developed MRONJ after surgical tooth extraction were on a drug holiday, therefore, drug holiday has no benefit or may even have a harmful effect on the development of MRONJ and the patient-reported health state

**Figure 2:**
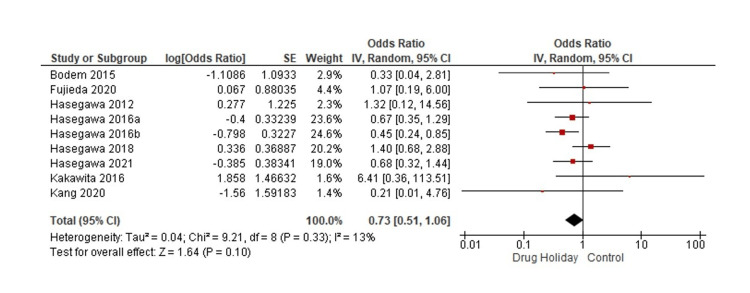
Forest plot of the association between the drug holiday group and non-drug holiday group and the development of MRONJ following tooth extraction MRONJ: medication-related osteonecrosis of the jaw.

The current meta-analysis included 4847 cases (drug holiday group) and 1961 controls (no drug holiday group). The randomized trial [20] was not included in the quantitative analysis with the observational studies to reduce the possible heterogeneity associated with different study designs. Based on the data of the observational studies, the pooled summary OR was 0.73 (95% CI: 0.51-1.06) in the random-effect model for the drug holiday group compared to the non-drug holiday group (Figure 2). In other words, the drug holiday group was not significantly different from the non-drug holiday group in the development of MRONJ following a tooth extraction procedure (p = 0.10). The study by Hasegawa et al. [15] had two independent samples based on the duration of the drug holiday and was included twice in the same forest plot. The statistical heterogeneity was low across all studies (I^2 ^= 13%, p = 0.33).

**Figure 3:**
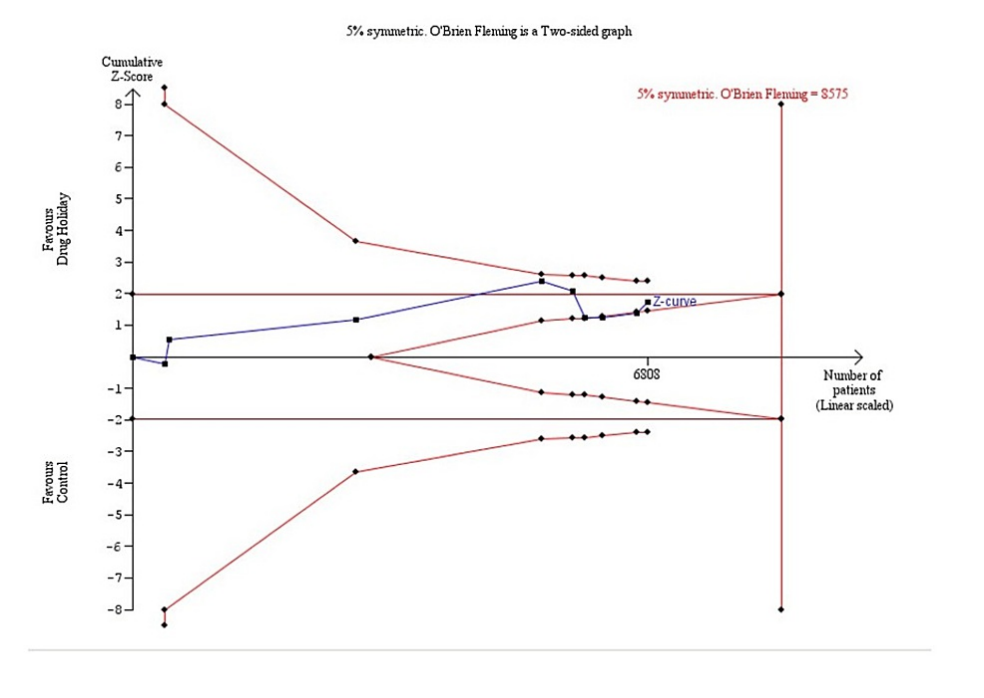
Trial sequential analysis (TSA) for the outcome

The resulting trial sequential analysis is shown in Figure 3. The cumulative number of patients included in the meta-analysis is represented in the x-axis. The y-axis represents the cumulative Z score. The required meta-analysis sample size was 8575 patients. After the fourth trial was added, the cumulative Z-statistic crossed above 1.96, which corresponds to the nominal threshold for statistical significance, using conventional techniques but did not cross the trial sequential boundary. From the fourth trial onwards, the meta-analysis was no longer nominally statistically significant. The last point of the Z-curve stays within the monitoring boundaries after new studies were added (Figure 3). The sample size did not exceed the required meta-analysis sample size. This meta-analysis was inconclusive and further studies are required.

Sensitivity analyses were performed for the route of administration, study design, primary disease, and the influence of individual study on the overall risk of MRONJ development. The estimated effects remained unchanged (Table 2). By sequentially removing the most recent trials and re-analyzing the remaining dataset, similar OR and 95% CI were obtained after the exclusion. This indicated a high degree of stability in the results.

**Table 2:** Subgroup meta-analyses of observational studies to explore sources of heterogeneity

Subgroups	n	OR (95% CI)	P-values for heterogeneity
Route of administration			
IV	2	0.98 (0.29, 3.32)	0.21
Oral	4	0.63 (0.35, 1.13)	0.27
Study design			
Prospective	2	0.61 (0.12, 3.02)	0.4
Retrospective	7	0.75 (0.49, 1.16)	0.21
Primary disease			
Osteoporosis	3	1.60 (0.45, 5.63)	0.57
Cancer	3	0.90 (0.47, 1.71)	0.25

In terms of the risk of bias assessment, all studies were graded as low risk (Table 3).

**Table 3:** Risk of bias assessment: Newcastle-Ottawa Quality Assessment Scale

No.	Reference	Was the exposed cohort representative?	Selection of the non-exposed cohort	Ascertainment of exposure	Demonstration that outcome of interest was not present at the start of the study	Comparability by design and analysis	Assessment of outcome	Was follow-up long enough for outcomes to occur?	Adequacy of follow-up of cohorts	Score
1	Bodem et al. (2015) [12]	*	*	*	*	**	*	*	*	9
2	Fujieda et al. (2020) [13]	*	*	*	*	**	*	*	*	9
3	Hasegawa et al. (2013) [16]	*	*	*	*	**	*	*	*	9
4	Hasegawa et al. (2017) (a & b) [15]	*	*	*	*	**	*	*	*	9
5	Hasegawa et al. (2019) [14]	*	*	*	*	*X	*	*	*	8
6	Hasegawa et al. (2021) [17]	*	*	*	*	*X	*	*	*	8
7	Kawakita et al. (2017) [19]	*	*	*	X	**	*	X	X	6
8	Kang et al. (2020) [18]	*	*	*	*	*X	*	*	*	8
9	Ottesen et al. (2022) [20]	*	*	*	*	**	*	*	*	9

The summary of the findings with the quality of the evidence (GRADE) assessment is shown in Table 4.

**Table 4:** GRADE assessment GRADE: Grading of Recommendations, Assessment, Development, and Evaluations; AR: antiresorptive; CI: confidence interval; OR: odds ratio.

Certainty assessment							No. of patients		Effect		Certainty	Importance
No. of studies	Study design	Risk of bias	Inconsistency	Indirectness	Imprecision	Other considerations	AR drug holiday	No drug holiday	Relative (95% CI)	Absolute (95% CI)		
MRONJ vs. No MRONJ												
9	Observational studies	Not serious	Not serious	Not serious	Serious	None	101/4847 (2.1%)	86/1961 (4.4%)	OR: 0.74 (0.51 to 1.06)	11 fewer per 1,000 (from 21 fewer to 3 more)	⨁◯◯◯ Very low	

Discussion

This study was the first meta-analysis to evaluate the efficacy of AR drug holidays on the risk of MRONJ following a tooth extraction procedure. As of now, only systematic reviews have been carried out to investigate this issue.

Our meta-analysis revealed the drug holiday group was not significantly different from the non-drug holiday group in the development of MRONJ following a tooth extraction procedure (p = 0.10). The statistical heterogeneity was low across all studies (I^2 ^= 13%, p = 0.33), based on the summarized results of the observational studies, with 4847 cases (drug holiday group) and 1961 controls (no drug holiday group). Also, our TSA findings demonstrated inconclusive evidence in support of the effect of drug holiday intervention. Nine studies fitted the PICO framework, concluding that drug holidays may not be implemented for bisphosphonate (BP) therapy.

Although most included studies did not directly compare discontinuation with the continuation of high-dose AR therapy, they conclude implementing drug holidays based on different observations. It is clear that the level of evidence was low but represents the best available information on this subject. Of all the included studies, only the one performed by Ottesen et al. used a randomized controlled design where patients with cancer receiving high-dose AR were randomized to a drug holiday from one month before to three months after surgical tooth extraction or drug continuation with a follow-up scheduled at one, three, and six months postoperatively. AR included denosumab and BP. Four denosumab patients from the drug holiday group developed MRONJ and the results indicated that a high-dose AR drug holiday does not prevent the development of MRONJ after surgical tooth extraction. Since this study utilized an interventional design, it was not included in the quantitative analysis with the observational studies although the strength of the study was very high [20].

BPs retained in the bone have a terminal half-life of many years [21], so discontinuing BP therapy for a short period of time as three months (a drug holiday) will have little impact on the BP already incorporated into the bone [22]. However, the overlying mucosa may recover more quickly if additional effects of these medications, such as their antiangiogenic action and inhibition of epithelial cell proliferation and migration, are decreased [23]. The longest investigated drug holiday was for three months, which found no significant differences between groups, and we were unable to definitively rule out the effectiveness of a drug holiday [16].

We performed sensitivity analyses to evaluate if the route of administration had an influence on the outcome. However, regardless of the route of administration, we did not find any significant difference between intravenous and oral AR holidays for the development of MRONJ. Previous studies have reported the incidence of MRONJ among patients receiving intravenous BP to be 0-51.9% [24]. On the other hand, some studies have shown that MRONJ incidence rates are low (0-2.8%) in patients receiving intravenous BP therapy after tooth extraction with new surgical procedures. However, only a few studies had clearly indicated the route of administration, hence further studies are warranted to come to a conclusion.

It was noted that none of the included papers investigated the relationship between high-cumulative and low-cumulative AR therapy and the development of MRONJ. BP and denosumab are administered at high IV frequency as part of treatment for malignancies, while they are administered at low frequency as part of treatment for osteoporosis. Some of these studies were mixed (e.g., including patients undergoing both high-dose and low-dose AR therapy), which also is a limitation difficult to avoid.

The health status of the patients in the included studies also varied. In a few studies, all the patients had been diagnosed with cancer [12,14], which means that the health of these patients was likely to be more compromised when compared to patients who did not have cancer (e.g., patients diagnosed with osteoporosis). A compromised immune response may increase susceptibility to infections and possibly MRONJ onset [17,25]. They may have other comorbidities such as diabetes, which will also contribute to lower immunity and susceptibility to infections. Fujieda et al. studied autoimmune disease in relation to the development of MRONJ and they revealed that patients with rheumatoid arthritis had a higher risk of developing osteonecrosis of the jaw (ONJ) [13]. This high evidence might also be explained due to the concomitant use of glucocorticoids with AR medications.

Another factor to consider is the duration of the AR treatment. The duration varied in the studies. A few studies did not describe the duration of AR agent treatment. Considering the strong correlation between AR treatment duration and MRONJ, this is a limitation of the study. Bodem et al. examined the risk of treatment failure in patients receiving IV BP therapy at the time of surgery compared with patients with previously completed or temporarily suspended intravenous BP therapy [12]. Their findings revealed that the patients currently undergoing intravenous BP therapy showed no higher risk for bisphosphonate-related osteonecrosis of the jaw (BRONJ) compared with patients who have paused or completed their intravenous BP therapy.

Tooth extraction is believed to be a major risk factor for the development of MRONJ [7,26]. Additionally, surgical procedural factors (such as open wounds, root amputations, and osteotomies) were significantly associated with MRONJ development [15]. Therefore, a previous BRONJ position paper from the Allied Task Force Committee of the Japanese Society for Bone and Mineral Research suggested that nonsurgical treatments should be performed rather than surgical treatments such as tooth extraction if dental treatments are desperately required [27].

Most of the excluded studies included study populations that were not homogeneous. A few studies did not have a comparison group [28-31]. Saia et al. reported that all patients included in their prospective cohort study had a drug holiday, meaning that no comparison was made between a drug holiday and “no drug holiday” [30]. Some of the studies had MRONJ diagnoses made at the time of the drug holidays [25,32-35]. Some used research methods that did not meet our inclusion criteria [33]. Using a national database, Jung et al. investigated the association between BP treatment and the occurrence of ONJ. Out of a total of 1569 patients included based on four-year retrospective periods, only 317 patients were being treated with high-dose BP therapy. It was found that 53.3% of all incidences of ONJ during the study period occurred after a drug holiday and that the frequency of ONJ occurrence rapidly decreased as the length of the drug holidays increased [36]. Some of these studies investigated the effect of discontinuation of BP therapy on the surgical outcome of MRONJ [25,32,33,35,37]. The reasons why studies were not included in the current review are available in Table 5.

**Table 5:** Characteristics of excluded studies BRONJ: bisphosphonate-related osteonecrosis of the jaw; BP: bisphosphonate; DRONJ: denosumab-related osteonecrosis of the jaw; ONJ: osteonecrosis of the jaw; MRONJ: medication-related osteonecrosis of the jaw.

Study	Author, year	Location	Design	Study population	Aim	Confounding factors	Reason for exclusion
Factors influencing the surgical treatment of bisphosphonate-related osteonecrosis of the jaws	Wutzl et al. (2012) [25]	Austria	Retrospective study	Patients treated for osteonecrosis of the jaw	To assess factors underlying the success of surgical treatment in patients with bisphosphonate-related osteonecrosis of the jaw (BRONJ)	Age, sex, primary disease, type of bisphosphonates, dosage of BP, drug holiday	Different populations and intervention
Long-term oral bisphosphonates delay healing after tooth extraction: a single institutional prospective study	Shudo et al. (2018) [28]	Japan	Prospective study	Patients who were receiving oral bisphosphonates for the prevention or treatment of osteoporosis and required tooth extraction	To evaluate the clinical safety of continuing oral bisphosphonate therapy in patients undergoing tooth extraction	Age, sex, reasons for BP therapy, primary disease, systemic risk factors: glucocorticoid administration, diabetes mellitus, rheumatoid arthritis, systemic lupus erythematosus, renal dialysis	No comparison group
MRONJ incidence after multiple teeth extractions in patients taking oral bisphosphonates without “drug holiday”: a retrospective chart review	Di Spirito et al. (2019) [29]	Italy	Retrospective study	Patients who were treated with oral BPs for osteoporosis for at least 3 years, who underwent multiple adjacent teeth extractions with a 12-month follow-up	To assess the occurrence of MRONJ after teeth extraction in patients taking oral bisphosphonates without a “drug holiday”	Gender, age, oral BP type, oral BP therapy duration	No comparison group
Occurrence of bisphosphonate-related osteonecrosis of the jaw after surgical tooth extraction	Saia et al. (2010) [30]	Italy	Prospective cohort study	Patients treated with nitrogen-containing bisphosphonates who underwent surgical tooth extraction with bone biopsy	To assess the incidence of and risk factors for BRONJ in patients who take nitrogen-containing BP and need a surgical tooth extraction	Age, cancer diagnosis, gender, baseline osteomyelitis	No comparison group
Denosumab-related osteonecrosis of the jaw: a retrospective study	Egloff-Juras et al. (2018) [31]	France	Retrospective study	Patients who were treated with denosumab	To evaluate the occurrence rate of denosumab-related osteonecrosis of the jaw (DRONJ) and to explore necrosis risk factors	Age, type of cancers, smoking, alcohol consumption, glucocorticoids or anti-angiogenic therapy, chemotherapy, diabetes, existence of denture, pressure sores, pre-therapeutic dental consultation	No drug holiday
Drug holiday as a prognostic factor of medication-related osteonecrosis of the jaw	Kim et al. (2014) [32]	Korea	Retrospective study	Patients diagnosed with MRONJ who visited the Department of Dentistry, Ajou University Hospital from May 2007 to March 2014	To identify post-treatment prognostic factors for MRONJ	Age, sex, primary disease, type of BP, type of surgical procedure	Different population
Bisphosphonate-induced avascular osteonecrosis of the jaws: a clinical report of 11 cases	Dimitrakopoulos et al. (2006) [33]	Greece	Retrospective study	Patients with necrotic bone lesions of the jaws of various extents	To evaluate the clinical characteristics of BRONJ and suggest a therapeutic protocol	None mentioned	Different populations, study design (case series)
Clinical characteristics and recurrence-related factors of medication-related osteonecrosis of the jaw	Kang et al. (2018) [34]	Korea	Retrospective study	Patients who were diagnosed with MRONJ	To investigate the demographic and clinical characteristics of patients with MRONJ and to assess factors affecting recurrence in surgical treatment	Type of drug, the duration of medication usage, route of administration, use of steroids, age, sex, systemic diseases	No comparison group, different population
Relevant factors for treatment outcome and time to healing in medication-related osteonecrosis of the jaws – a retrospective cohort study	Martins et al. (2017) [35]	Portugal	Retrospective cohort study	Patients diagnosed with MRONJ	To describe the characteristics of a population of patients with MRONJ and the factors associated with favorable outcomes. Also to Identify a temporal correlation between discontinuation of antiresorptive and healing time	Gender, age at diagnosis, primary disease, administered drug, route of administration, length of administration, time of discontinuation of antiresorptive medication, chronic steroid therapy, denture wear, local invasive procedures previous to MRONJ, and several co-morbidities	Different population: patients already diagnosed with MRONJ. Antiresorptive medication discontinuation contributes to reducing healing time in MRONJ
Drug holiday patterns and bisphosphonate-related osteonecrosis of the jaw	Jung et al. (2019) [36]	Korea	Retrospective, cross-sectional study	Patients newly diagnosed with ONJ	To investigate the population-based patterns of the gaps between BP discontinuation and ONJ diagnosis	The use of corticosteroids, duration of treatment with Bisphosphonate, sex, age, co-morbidities (cancer diabetes), extractions	No comparison group
Clinical course and therapeutic outcomes of operatively and non-operatively managed patients with denosumab-related osteonecrosis of the jaw (DRONJ)	Hoefert et al. (2017) [37]	Germany	Retrospective study	Patients presented for evaluation and management of DRONJ between October 2010 and January 2016	To examine the clinical characteristics and operative and non-operative therapeutic outcomes in patients with DRONJ not previously exposed to other antiresorptives	Demographics, primary disease diagnosis, denosumab regimen and schedule, duration of therapy, concurrent primary disease therapy	Different Population
Dental treatments, tooth extractions, and osteonecrosis of the jaw in Japanese patients with rheumatoid arthritis: results from the IORRA cohort study	Furuya et al. (2017) [38]	Japan	Cross-sectional study	Patients who were diagnosed with rheumatoid arthritis	To evaluate dental treatments, tooth extractions, and osteonecrosis of the jaw (ONJ) in Japanese patients with rheumatoid arthritis (RA)	Sociodemographic measures, dental treatment, tooth extraction, medications, steroid usage	Self-reported study
Factors predicting the prognosis of oral alendronate-related osteonecrosis of the jaws: a 4-year cohort study	Lee et al. (2013) [39]	Taiwan	Retrospective cohort study	Patients who were treated for osteonecrosis of the jaw due to alendronate use	To assess the prognostic values of clinical variables and serum markers of bone turnover	Clinical history, age, sex, medical comorbidities, drug history, smoking, alcohol drinking, types of osteoporosis, pre-disposing, events related to bone necrosis, duration of alendronate use, duration of drug holiday	Different population
Temporal trends and factors associated with bisphosphonate discontinuation and restart	Adami et al. (2020) [40]	United States	Retrospective cohort study	Female patients who were treated with bisphosphonates for more than three years	To investigate temporal trends of bisphosphonate discontinuation and identified factors associated with discontinuation and restart of osteoporosis therapy	NA	Different study objective
Long waiting time before tooth extraction may increase delayed wound healing in elderly Japanese	Kamimura et al. (2019) [41]	Japan	Cross-sectional study	Patients aged ≥ 60 years who visited our clinics or hospitals from September 2016 to May 2017 were invited to answer a structured questionnaire	To explore the agreement between long waiting time before tooth extraction and delayed wound healing after tooth extraction regardless of the use of BP	Sex, age, height, weight, self-reported kyphosis, duration before tooth extraction, number of teeth lost, self-reported periodontal condition, frequency of tooth brushing, smoking, diabetes mellitus, hypertension, rheumatoid arthritis, steroid use, use and duration of use of bisphosphonate and/or denosumab, delayed wound healing longer than 8 weeks after a tooth extraction during the past year	Different study objective
The incidence of medication-related osteonecrosis of the jaw following tooth extraction in patients prescribed oral bisphosphonates	Barry et al. (2021) [42]	United Kingdom	Retrospective study	Patients on oral bisphosphonate who had extractions over an eight-year period	To investigate the incidence of MRONJ following tooth extraction in patients taking oral bisphosphonates	Sex, medical comorbidities, duration of therapy, site of extraction	Different objective

The strengths of this meta-analysis include the use of cumulative meta-analysis and the TSA. We conducted an exhaustive literature search across a number of databases including PubMed, Embase, Cochrane Library, Web of Science, and Scopus to obtain a summary measure of the association between BP drug holiday and MRONJ. This reduced the risk of missing studies that could have resulted in selection bias. Another advantage of the current analysis is the inclusion of a greater number of studies and participants from nine studies. The confirmation of the stability of the results through the sensitivity analysis and the high quality of the included studies (NCO) is another strength of the study removing potential differences by subgroup analysis.

One of the limitations of the study was that the studies included were mostly retrospective and non-randomized and non-matched. Large-scale, prospective cohort studies are needed to evaluate predictors of MRONJ in patients who are on AR holidays following tooth extraction. The majority of the research used relatively small sample sizes, which resulted in low-grade scientific evidence. Even though it is very simple to establish an important research topic, it is challenging to execute randomized controlled trials or controlled prospective studies with enough participants to provide a response because of the low incidence of MRONJ and substantial patient and AR therapy variance. It is also challenging to circumvent this issue because some of these studies were mixed (e.g., some studies included patients receiving both high-dose and low-dose AR therapy), some had diverse delivery methods, and the health status of the patients differed. The studies included were observational, and as such, they had inherent bias constraints.

However, due to the significant level of imprecision, the quality of the GRADE assessment's evidence for the evaluated outcome was low. Hence, the results of this meta-analysis should be evaluated with caution.

## Conclusions

The effectiveness of high-dose AR drug holidays is still a matter of debate. The results indicate that a high-dose AR drug holiday does not prevent the development of MRONJ after a tooth extraction procedure. Large prospective studies and high-quality randomized trials may be able to provide more conclusive results, but they are challenging to conduct because of the unexpected outcomes and scarcity of qualified patients.

## References

[1] AlDhalaan NA, BaQais A, Al-Omar A (2020). Medication-related osteonecrosis of the jaw: a review. Cureus.

[2] Ruggiero SL, Dodson TB, Aghaloo T, Carlson ER, Ward BB, Kademani D (2022). American Association of Oral and Maxillofacial Surgeons' position paper on medication-related osteonecrosis of the jaws—2022 update. J Oral Maxillofac Surg.

[3] Beth-Tasdogan NH, Mayer B, Hussein H, Zolk O (2017). Interventions for managing medication-related osteonecrosis of the jaw. Cochrane Database Syst Rev.

[4] Otto S, Pautke C, Van den Wyngaert T, Niepel D, Schiødt M (2018). Medication-related osteonecrosis of the jaw: prevention, diagnosis and management in patients with cancer and bone metastases. Cancer Treat Rev.

[5] Filleul O, Crompot E, Saussez S (2010). Bisphosphonate-induced osteonecrosis of the jaw: a review of 2,400 patient cases. J Cancer Res Clin Oncol.

[6] Yazdi PM, Schiodt M (2015). Dentoalveolar trauma and minor trauma as precipitating factors for medication-related osteonecrosis of the jaw (ONJ): a retrospective study of 149 consecutive patients from the Copenhagen ONJ Cohort. Oral Surg Oral Med Oral Pathol Oral Radiol.

[7] Ruggiero SL, Dodson TB, Fantasia J, Goodday R, Aghaloo T, Mehrotra B, O'Ryan F (2014). American Association of Oral and Maxillofacial Surgeons position paper on medication-related osteonecrosis of the jaw—2014 update. J Oral Maxillofac Surg.

[8] Khan AA, Morrison A, Kendler DL (2017). Case-based review of osteonecrosis of the jaw (ONJ) and application of the international recommendations for management from the International Task Force on ONJ. J Clin Densitom.

[9] Svejda B, Muschitz Ch, Gruber R (2016). Position paper on medication-related osteonecrosis of the jaw (MRONJ). (Article in German). Wien Med Wochenschr.

[10] Snowden JA, Ahmedzai SH, Ashcroft J (2011). Guidelines for supportive care in multiple myeloma 2011. Br J Haematol.

[11] Ottesen C, Schiodt M, Gotfredsen K (2020). Efficacy of a high-dose antiresorptive drug holiday to reduce the risk of medication-related osteonecrosis of the jaw (MRONJ): a systematic review. Heliyon.

[12] Bodem JP, Kargus S, Eckstein S, Saure D, Engel M, Hoffmann J, Freudlsperger C (2015). Incidence of bisphosphonate-related osteonecrosis of the jaw in high-risk patients undergoing surgical tooth extraction. J Craniomaxillofac Surg.

[13] Fujieda Y, Doi M, Asaka T (2020). Incidence and risk of antiresorptive agent-related osteonecrosis of the jaw (ARONJ) after tooth extraction in patients with autoimmune disease. J Bone Miner Metab.

[14] Hasegawa T, Hayashida S, Kondo E (2019). Medication-related osteonecrosis of the jaw after tooth extraction in cancer patients: a multicenter retrospective study. Osteoporos Int.

[15] Hasegawa T, Kawakita A, Ueda N (2017). A multicenter retrospective study of the risk factors associated with medication-related osteonecrosis of the jaw after tooth extraction in patients receiving oral bisphosphonate therapy: can primary wound closure and a drug holiday really prevent MRONJ?. Osteoporos Int.

[16] Hasegawa T, Ri S, Umeda M (2013). The observational study of delayed wound healing after tooth extraction in patients receiving oral bisphosphonate therapy. J Craniomaxillofac Surg.

[17] Hasegawa T, Ueda N, Yamada SI (2021). Denosumab-related osteonecrosis of the jaw after tooth extraction and the effects of a short drug holiday in cancer patients: a multicenter retrospective study. Osteoporos Int.

[18] Kang SH, Park SJ, Kim MK (2020). The effect of bisphosphonate discontinuation on the incidence of postoperative medication-related osteonecrosis of the jaw after tooth extraction. J Korean Assoc Oral Maxillofac Surg.

[19] Kawakita A, Yanamoto S, Morishita K (2017). Discontinuing oral bisphosphonate therapy during dental extraction does not prevent osteonecrosis of the jaw: a multicenter retrospective study of 341 patients with propensity score matching analysis. J Oral Maxillofac Surg Med Pathol.

[20] Ottesen C, Schiodt M, Jensen SS, Kofod T, Gotfredsen K (2022). Tooth extractions in patients with cancer receiving high-dose antiresorptive medication: a randomized clinical feasibility trial of drug holiday versus drug continuation. Oral Surg Oral Med Oral Pathol Oral Radiol.

[21] Ensrud KE, Barrett-Connor EL, Schwartz A (2004). Randomized trial of effect of alendronate continuation versus discontinuation in women with low BMD: results from the Fracture Intervention Trial long-term extension. J Bone Miner Res.

[22] Van den Wyngaert T, Huizing MT, Fossion E, Vermorken JB (2009). Bisphosphonates in oncology: rising stars or fallen heroes. Oncologist.

[23] Migliorati CA (2003). Bisphosphanates and oral cavity avascular bone necrosis. J Clin Oncol.

[24] Gaudin E, Seidel L, Bacevic M, Rompen E, Lambert F (2015). Occurrence and risk indicators of medication-related osteonecrosis of the jaw after dental extraction: a systematic review and meta-analysis. J Clin Periodontol.

[25] Wutzl A, Pohl S, Sulzbacher I (2012). Factors influencing surgical treatment of bisphosphonate-related osteonecrosis of the jaws. Head Neck.

[26] Vahtsevanos K, Kyrgidis A, Verrou E (2009). Longitudinal cohort study of risk factors in cancer patients of bisphosphonate-related osteonecrosis of the jaw. J Clin Oncol.

[27] Yoneda T, Hagino H, Sugimoto T (2010). Bisphosphonate-related osteonecrosis of the jaw: position paper from the Allied Task Force Committee of Japanese Society for Bone and Mineral Research, Japan Osteoporosis Society, Japanese Society of Periodontology, Japanese Society for Oral and Maxillofacial Radiology, and Japanese Society of Oral and Maxillofacial Surgeons. J Bone Miner Metab.

[28] Shudo A, Kishimoto H, Takaoka K, Noguchi K (2018). Long-term oral bisphosphonates delay healing after tooth extraction: a single institutional prospective study. Osteoporos Int.

[29] Di Spirito F, Argentino S, Martuscelli R, Sbordone L (2019). MRONJ incidence after multiple teeth extractions in patients taking oral bisphosphonates without "drug holiday": a retrospective chart review. Oral Implant.

[30] Saia G, Blandamura S, Bettini G (2010). Occurrence of bisphosphonate-related osteonecrosis of the jaw after surgical tooth extraction. J Oral Maxillofac Surg.

[31] Egloff-Juras C, Gallois A, Salleron J, Massard V, Dolivet G, Guillet J, Phulpin B (2018). Denosumab-related osteonecrosis of the jaw: a retrospective study. J Oral Pathol Med.

[32] Kim YH, Lee HK, Song SI, Lee JK (2014). Drug holiday as a prognostic factor of medication-related osteonecrosis of the jaw. J Korean Assoc Oral Maxillofac Surg.

[33] Dimitrakopoulos I, Magopoulos C, Karakasis D (2006). Bisphosphonate-induced avascular osteonecrosis of the jaws: a clinical report of 11 cases. Int J Oral Maxillofac Surg.

[34] Kang MH, Lee DK, Kim CW, Song IS, Jun SH (2018). Clinical characteristics and recurrence-related factors of medication-related osteonecrosis of the jaw. J Korean Assoc Oral Maxillofac Surg.

[35] Martins AS, Correia JA, Salvado F, Caldas C, Santos N, Capelo A, Palmela P (2017). Relevant factors for treatment outcome and time to healing in medication-related osteonecrosis of the jaws - a retrospective cohort study. J Craniomaxillofac Surg.

[36] Jung SY, Suh HS, Park JW, Kwon JW (2019). Drug holiday patterns and bisphosphonate-related osteonecrosis of the jaw. Oral Dis.

[37] Hoefert S, Yuan A, Munz A, Grimm M, Elayouti A, Reinert S (2017). Clinical course and therapeutic outcomes of operatively and non-operatively managed patients with denosumab-related osteonecrosis of the jaw (DRONJ). J Craniomaxillofac Surg.

[38] Furuya T, Maeda S, Momohara S, Taniguchi A, Yamanaka H (2017). Dental treatments, tooth extractions, and osteonecrosis of the jaw in Japanese patients with rheumatoid arthritis: results from the IORRA cohort study. J Bone Miner Metab.

[39] Lee JJ, Cheng SJ, Wang JJ, Chiang CP, Chang HH, Chen HM, Kok SH (2013). Factors predicting the prognosis of oral alendronate-related osteonecrosis of the jaws: a 4-year cohort study. Head Neck.

[40] Adami G, Jaleel A, Curtis JR (2020). Temporal trends and factors associated with bisphosphonate discontinuation and restart. J Bone Miner Res.

[41] Kamimura M, Taguchi A, Komatsu M (2019). Long waiting time before tooth extraction may increase delayed wound healing in elderly Japanese. Osteoporos Int.

[42] Barry E, Taylor T, Patel J, Hamid U, Bryant C (2021). The incidence of medication-related osteonecrosis of the jaw following tooth extraction in patients prescribed oral bisphosphonates. [PREPRINT]. Br Dent J.

